# Evaluation of the effects on the QT-interval of 4 artemisinin-based combination therapies with a correction-free and heart rate-free method

**DOI:** 10.1038/s41598-018-37113-5

**Published:** 2019-01-29

**Authors:** Christian Funck-Brentano, Nouhoum Ouologuem, Stephan Duparc, Mathieu Felices, Sodiomon B. Sirima, Issaka Sagara, Issiaka Soulama, Jean-Bosco Ouedraogo, Abdoul H. Beavogui, Isabelle Borghini-Fuhrer, Yasmin Khan, Abdoulaye A. Djimdé, Pascal Voiriot

**Affiliations:** 1 INSERM, CIC-1421 and UMR ICAN 1166, Sorbonne Université, Faculty of Medicine, AP-HP, Pitié-Salpêtrière Hospital, Department of Pharmacology and Clinical Investigation Center, Institute of Cardiometabolism and Nutrition (ICAN), F-75013 Paris, France; 2 Malaria research and training center. Département d’épidémiologie des affections parasitaires, Faculté de médecine de pharmacie et d’odonto-stomatologie. P.O. Box 1805, Point G, Bamako, Mali; 3 Medicines for Malaria Venture, International Center Cointrin, 20 route de Pré-Bois, 1215 Geneva 15, Switzerland; 4 Phinc Development, Immeuble Genavenir 8, 5 rue Henri Desbruères, 91030 Evry Cedex, France; 5 Centre national de recherche et de formation sur le paludisme, 01 P.O. Box 2208, Ouagadougou 01, Burkina Faso; 6 IRSS, Direction Régionale de l’Ouest, 399, Avenue de la Liberté 01, P.O. Box 545, Bobo-Dioulasso 01, Burkina Faso; 7 Centre National de Formation et de Recherche en Santé Rurale de Mafèrinyah, P.O. Box 2649, Conakry, Guinea; 8 Cardiabase, 84 avenue du XXème Corps, 54000 Nancy, France

## Abstract

Several antimalarial drugs are known to prolong ventricular repolarization as evidenced by QT/QTc interval prolongation. This can lead to Torsades de Pointes, a potentially lethal ventricular arrhythmia. Whether this is the case with artemisinin-based combination therapies (ACTs) remains uncertain. Assessment of the extent of QTc prolongation with antimalarials is hampered by important variations of heart rate during malaria crises and previous studies have reported highly variable values of QTc prolongations with ACTs. We assessed QTc prolongation with four ACTs, using high quality ECG recording and measurement techniques, during the first episode of malaria in 2,091 African patients enrolled in the WANECAM study which also monitored clinical safety. Using an original and robust method of QTc assessment, independent from heart rate changes and from the method of QT correction, we were able to accurately assess the extent of mean maximum QTc prolongation with the four ACTs tested. There was no evidence of proarrhythmia with any treatment during the study although dihydroartemisinin-piperaquine, artesunate-amodiaquine and artemether-lumefantrine significantly prolonged QTc. The extent of prolongation of ventricular repolarization can be accurately assessed in studies where heart rate changes impede QTc assessment.

## Introduction

According to the World Health Organization, the number of malaria cases fell from an estimated 262 million in 2000 to 216 million in 2016. The number of fatal cases showed an even more spectacular decline from an estimated 839,000 in 2000 to 445,000 in 2016^1^. Although the incidence of malaria cases has fallen globally since 2010, the rate of decline has stopped and even reversed in some regions since 2014. Mortality rates have followed a similar pattern. Prevention measures such as the use of mosquito nets and increased access to effective treatments contributed to this success^1^. To counter the threat of resistance of *P. falciparum* to monotherapies, and to improve treatment outcome, the World Health Organization has recommended that artemisinin-based combination therapies (ACT) be used for the treatment of uncomplicated *P. falciparum* malaria^2^.

Previous studies with artesunate-amodiaquine (ASAQ) and/or artemether-lumefantrine (AL) have shown that these therapies are well tolerated and efficacious^3–6^. However, resistance to artemisinin has been reported^7,8^ in the greater Mekong region necessitating the need for the development of alternative ACTs. Pyronaridine-artesunate (PA) and dihydroartemisinin-piperaquine (DHA-PQP) are such therapies, which were recently approved in Europe^9^. Rather than using only one ACT, countries could better use several ACTs available to them in order to reduce the risk of resistance development and keep these treatments efficacious as long as possible.

Several antimalarial drugs are known to prolong ventricular repolarization as evidenced by QT/QTc interval prolongation. QT/QTc prolongation is associated with rare cases of Torsades de Pointes, a cardiac arrhythmia which is often self-terminating but can lead to ventricular fibrillation and sudden death. Prolongation of the QT interval is therefore used as a surrogate marker for possible effects of drugs on ventricular rhythm. However, this parameter is very sensitive to changes in heart rate and hence different correction methods, such as Bazett’s and Fridericia’s corrections, have been proposed to normalize QT to a heart rate of 60 beats per minute^10^. Malaria represents a particularly difficult case with respect to correcting the QT interval for changes in heart rate and highly variable QTc prolongation values have been reported in studies of antimalarial drugs^11–14^. Following efficacious treatment of typically anxious patients, who are often young children, heart rate decreases as a result of fever and anxiety regression. This heart rate change, which is due to regression of malaria symptoms rather than to the administered drugs, makes it difficult to properly assess the effect of antimalarial drugs on QTc interval duration during therapeutic use.

The present report describes the ECG results obtained during the first episode of malaria in the WANECAM study, registered at the Pan African Clinical Trials Registry under the number PACTR201105000286876, during which high quality ECGs were collected as part of the safety evaluations. Results from the WANECAM study have recently been published^15^. Particular emphasis was put on the type of QT interval correction to be used and a new approach is proposed on how to assess the effects of drugs on QT interval duration when measurements are made at very different heart rates before and during drug exposure, a situation which is encountered with many drugs other than antimalarial drugs.

## Results

### Study population

ECG recordings were received in a total of 2,660 patients. Due to missing baseline or post-dose recordings, inconsistent visit identification or duplicate evaluations, 569 patients were excluded from the analyses. A summary of demographic data of the remaining 2,091 patients is provided in Table 1. A similar number of male and female patients participated. The majority of patients (74.1%) were aged 11 years or younger of whom 2.1% were younger than 2 years. The distribution of patients in the different age categories was roughly similar amongst ACTs.

**Table 1:** Demographic characteristics of the study population included in the analyses by treatment and country.

			DHA-PQP	PA	ASAQ	AL	All
Age		N	701	556	412	422	2091
		Mean (range)	8.6 (0–71)	10.5 (1–62)	7.9 (0–43)	9.8 (0–64)	9.2 (0–71)
	<2 years	N (%)	18 (2.6)	7 (1.3)	11 (2.7)	8 (1.9)	44 (2.1)
	2–11 years	N (%)	535 (76.3)	361 (64.9)	323 (78.4)	286 (67.8)	1505 (72.0)
	12–18 years	N (%)	122 (17.4)	127 (22.8)	68 (16.5)	99 (23.5)	416 (19.9)
	>18 years	N (%)	26 (3.7)	61 (11.0)	10 (2.4)	29 (6.9)	126 (6.0)
Gender	Female	N (%)	342 (48.8)	287 (51.6)	187 (45.4)	208 (49.3)	1024 (49.0)
	Male	N (%)	359 (51.2)	269 (48.4)	225 (54.6)	214 (50.7)	1067 (51.0)
Country	Burkina Faso	N (%)	277 (39.5)	198 (35.6)	124 (30.1)	213 (50.5)	812 (38.8)
	Guinea	N (%)	134 (19.1)	89 (16.0)	126 (30.6)		349 (16.7)
	Mali	N (%)	290 (41.4)	269 (48.4)	162 (39.3)	209 (49.5)	930 (44.5)

DHA-PQP, dihydroartemisinin-piperaquine; PA, pyronaridine-artesunate; ASAQ, artesunate-amodiaquine; AL, artemether-lumefantrine.

### Effect of study treatments on ECG parameters other than QTc

Baseline values for heart rate, RR, PR, QRS and QT were similar in the 4 treatment groups although heart rate on average tended to be lower (and RR interval longer) in the PA group when compared to the 3 other groups. On the 3^rd^ day of treatment administration a marked decrease in heart rate was observed, which was slightly less in the PA group when compared to the other ACTs. Small increases in PR and QRS intervals were noted without any major difference between ACTs. In all 4 treatment groups, QT interval prolonged as expected with the decrease of heart rate. This was most pronounced in the DHA-PQP and ASAQ groups and least pronounced in PA-treated patients (Table 2).

**Table 2:** Baseline and change from baseline for heart rate and RR, PR, QRS and QT intervals by study treatment.

Treatment	Heart rate (bpm)	RR (ms)	PR (ms)	QRS (ms)	QT (ms)
**Baseline**					
DHA-PQP	108.8 (21.5)	575.3 (127.3)	133.1 (20.4)	77.1 (8.7)	318.6 (33.7)
PA	105.8 (22.6)	595.4 (137.1)	135.3 (17.9)	76.9 (8.2)	321.1 (36.5)
ASAQ	109.3 (22.0)	573.4 (125.7)	132.7 (17.6)	76.0 (8.8)	318.3 (33.7)
AL	109.0 (22.0)	575.0 (125.8)	133.1 (19.2)	77.1 (7.8)	317.5 (33.3)
**Change from baseline**					
DHA-PQP	−23.7 (−24.8; −22.6)	154.6 (147.5; 161.7)	10.7 (9.8; 11.6)	2.9 (2.5; 3.4)	54.6 (52.5; 56.6)
PA	−19.2 (−20.4; −17.9)	127.0 (118.2; 135.9)	8.1 (7.1; 9.1)	2.6 (2.1; 3.0)	29.4 (27.2; 31.6)
ASAQ	−27.8 (−29.2; −26.4)	191.0 (181.4; 200.6)	13.1 (12.0; 14.3)	6.5 (5.8; 7.1)	60.2 (57.6; 62.7)
AL	−25.5 (−26.9; −24.0)	170.5 (160.8; 180.3)	10.5 (9.2; 11.9)	2.5 (1.9; 3.1)	45.9 (43.4; 48.3)

Data are expressed as mean (SD for baseline and 90% confidence interval for change from baseline). DHA-PQP, dihydroartemisinin-piperaquine; PA, pyronaridine-artesunate; ASAQ, artesunate-amodiaquine; AL, artemether-lumefantrine.

### Effect of study treatments on QTc

Baseline values for QTcF, QTcB and QTcSS were similar in the 4 treatment groups. Irrespective of the correction method, treatment with DHA-PQP and ASAQ resulted in a prolongation of the QTc interval. However, the magnitude of the effect varied from 31.3 ms for QTcF to 16.3 ms for QTcB with DHA-PQP and from 30.9 ms for QTcF to 12.5 ms for QTcB with ASAQ. Treatment with PA and AL did not affect QTc when considering QTcB and QTcSS. In contrast, both treatments increased QTcF but to a lesser extent than DHA-PQP and ASAQ. For all treatments, changes from baseline were the largest for QTcF, the smallest for QTcB and with intermediate values for QTcSS (Table 3).

**Table 3:** Baseline and change from baseline in QTc by correction method and by study treatment.

Treatment	QTcF (ms)	QTcB (ms)	QTcSS (ms)
**Baseline**			
DHA-PQP	384.3 (21.0)	422.9 (21.3)	415.5 (21.1)
PA	383.0 (21.4)	419.2 (20.3)	411.8 (20.6)
ASAQ	384.4 (20.8)	423.2 (20.8)	415.5 (20.4)
AL	383.1 (21.0)	421.7 (22.0)	414.3 (21.4)
**Change from baseline**			
DHA-PQP	31.3 (29.7; 32.8)	16.3 (14.8; 17.9)	19.4 (17.9; 21.0)
PA	9.2 (7.8; 10.6)	−3.7 (−5.1; −2.3)	−0.8 (−2.2; 0.6)
ASAQ	30.9 (29.0; 32.7)	12.5 (10.5; 14.5)	16.2 (14.3; 18.1)
AL	18.8 (29.0; 32.7)	1.8 (−0.1; 3.6)	5.2 (3.4; 7.0)

Data are expressed as mean (SD for baseline and 90% confidence interval for change from baseline). DHA-PQP, dihydroartemisinin-piperaquine; PA, pyronaridine-artesunate; ASAQ, artesunate-amodiaquine; AL, artemether-lumefantrine.

**Figure 1:**
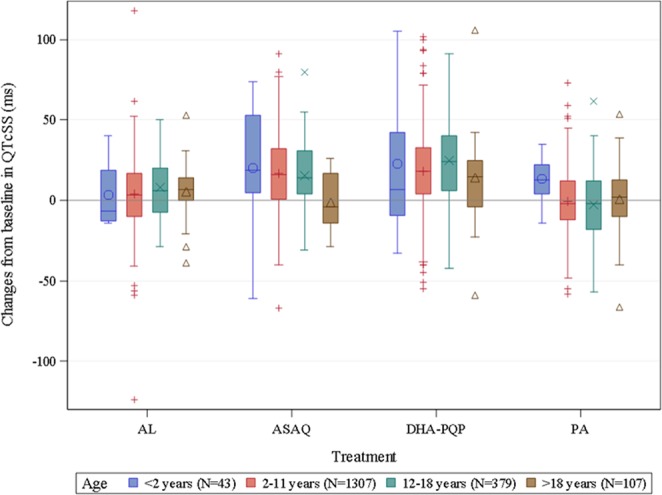
The influence of age on the effect of dihydroartemisinin-piperaquine (DHA-PQP), pyronaridine-artesunate (PA), artesunate-amodiaquine (ASAQ) and artemether-lumefantrine (AL) on the QTcSS interval. Boxes represent the 25th (Q1) and 75th (Q3) percentiles. Horizontal lines and symbols inside the boxes, represent medians and means, respectively. The ends of the whiskers represent the 1.5 interquartile range of the upper and lower quartiles. Symbols above or below the whiskers represent outliers.

Depending on age category and sex, correction exponents for QTcSS varied from 0.423 to 0.523. The influence of age on the effects of the 4 investigated ACTs was explored graphically as illustrated in Fig. 1. No statistically significant difference between the age categories 2 to 11 years and 12 to 18 years were noted.

### Adequacy of QT correction methods

**Figure 2:**
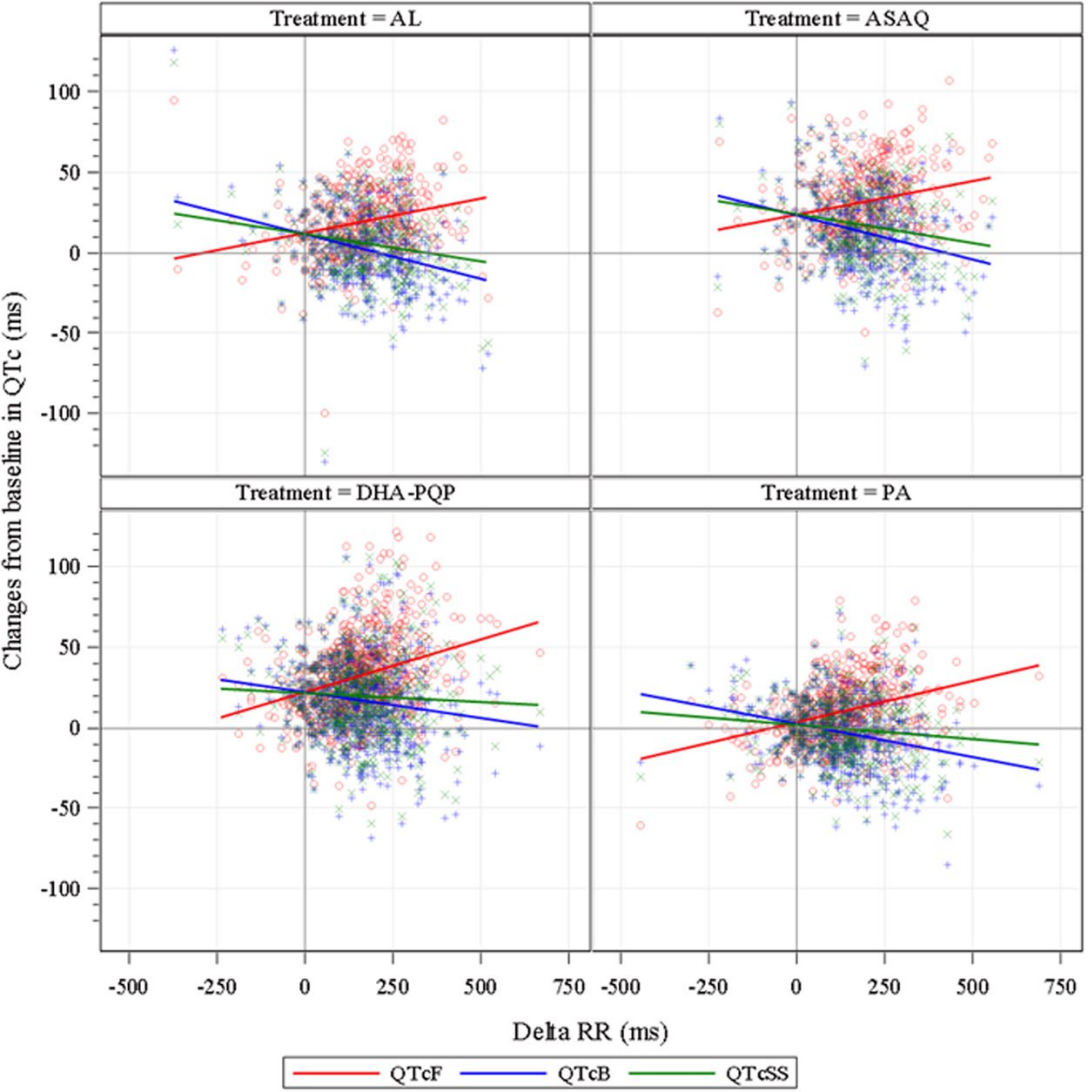
Individual values for the correlation between the changes from baseline in RR interval and the QT/QTc interval by treatment. The lines represent the regression curves of the modeling of the QT/QTc-RR relationship. DHA-PQP, dihydroartemisinin-piperaquine; PA, pyronaridine-artesunate; ASAQ, artesunate-amodiaquine; AL, artemether-lumefantrine.

The relationship between the changes of RR and QTc interval (ΔQTc vs. ΔRR regressions) from baseline for each correction method is shown in Fig. 2. For none of the tested correction methods was a horizontal regression line obtained, i.e. no QT correction method fully accounted for heart rate variations. QTcF increased as RR interval increased whereas QTcB and QTcSS decreased as RR interval increased. In order to determine the average effect of study treatments on QT interval independently from any heart rate correction, i.e., when the change in RR from baseline was 0, the intercept of the regression line of the ΔQTc versus ΔRR relationship was estimated. Results are presented in Table 4 and indicate that this method of assessment of QT changes yields consistent results irrespective of the biases introduced by all QT correction formulae and represents a correction-free and heart rate-free assessment of drug effect on the duration of ventricular repolarization. The results show the absence of QTc prolongation with PA and confirm the QTc interval prolongation with DHA-PQP, ASAQ and AL. Prolongation of the QT interval with AL, however, is smaller than with DHA-PQP and ASAQ.

**Table 4:** Change from baseline (90% confidence interval) in the absence of a change in RR (RR0) estimated from the regression line from change from baseline in QTc versus change from baseline in RR interval for each correction method by treatment.

Treatment	QTcF (ms)	QTcB (ms)	QTcSS (ms)
DHA-PQP	21.1 (18.8, 23.4)	21.3 (19.0, 23.7)	21.1 (18.7, 23.4)
PA	2.7 (0.5, 4.8)	1.6 (−0.7, 3.8)	1.5 (−0.7, 3.7)
ASAQ	23.0 (19.6, 26.3)	23.0 (19.5, 26.5)	23.0 (19.5, 26.5)
AL	11.7 (8.7, 14.7)	11.2 (8.0, 14.3)	11.0 (7.9, 14.1)

DHA-PQP, dihydroartemisinin-piperaquine; PA, pyronaridine-artesunate; ASAQ, artesunate-amodiaquine; AL, artemether-lumefantrine.

### Categorical analysis

Table 5 summarizes the results of the categorical analysis. When compared to QTcB and QTcSS, fewer absolute abnormal QTcF values were recorded at baseline and after treatment with an ACT. In contrast, abnormal changes from baseline were more frequent when QT was corrected using Fridericia’s method when compared to the other two correction methods. Irrespective of the correction method, abnormal QTc values (both absolute and change from baseline) were more frequently observed in patients treated with DHA-PQP and ASAQ than in patients treated with PA with intermediate results obtained with AL. QTc values > 500 ms were rarely recorded in this study. However, in >11% of patients treated with DHA-PQP and ASAQ, an increase from baseline in QTcF exceeding 60 ms was recorded (Table 5).

**Table 5:** Summary of abnormal values of QTc by correction method.

	DHA-PQP (N = 701)		PA (N = 556)		ASAQ (N = 412)		AL (N = 422)	
	Baseline	Day 3	Baseline	Day 3	Baseline	Day 3	Baseline	Day 3
**QTcF**								
450 < QTcF ≤ 480 ms	0 (0.0)	36 (5.1)	2 (0.4)	0 (0.0)	2 (0.5)	17 (4.1)	0 (0.0)	0 (0.0)
480 < QTcF ≤ 500 ms	0 (0.0)	9 (1.3)	0 (0.0)	0 (0.0)	0 (0.0)	2 (0.5)	1 (0.2)	0 (0.0)
QTcF > 500 ms	0 (0.0)	4 (0.6)	0 (0.0)	0 (0.0)	0 (0.0)	1 (0.2)	0 (0.0)	0 (0.0)
30 < ΔQTcF ≤ 60 ms		262 (37.4)		67 (12.1)		151 (36.7)		101 (23.9)
ΔQTcF > 60 ms		79 (11.3)		9 (1.6)		48 (11.7)		12 (2.8)
**QTcB**								
450 < QTcB ≤ 480 ms	67 (9.6)	166 (23.7)	23 (4.1)	20 (3.6)	33 (8.0)	79 (19.2)	21 (5.0)	39 (9.2)
480 < QTcB ≤ 500 ms	2 (0.3)	29 (4.1)	3 (0.5)	1 (0.2)	2 (0.5)	14 (3.4)	1 (0.2)	2 (0.5)
QTcB > 500 ms	0 (0.0)	12 (1.7)	0 (0.0)	0 (0.0)	0 (0.0)	2 (0.5)	1 (0.2)	0 (0.0)
30 < ΔQTcB ≤ 60 ms		144 (20.5)		22 (4.0)		81 (19.7)		43 (10.2)
ΔQTcB > 60 ms		33 (4.7)		1 (0.2)		14 (3.4)		2 (0.5)
**QTcSS**								
450 < QTcSS ≤ 480 ms	38 (5.4)	133 (19.0)	11 (2.0)	12 (2.2)	15 (3.6)	65 (15.8)	7 (1.7)	29 (6.9)
480 < QTcSS ≤ 500 ms	1 (0.1)	22 (3.1)	2 (0.4)	0 (0.0)	1 (0.2)	5 (1.2)	0 (0.0)	1 (0.2)
QTcSS > 500 ms	0 (0.0)	11 (1.6)	0 (0.0)	0 (0.0)	0 (0.0)	3 (0.7)	1 (0.2)	0 (0.0)
30 < ΔQTcSS ≤ 60 ms		176 (25.1)		32 (5.8)		95 (23.1)		51 (12.1)
ΔQTcSS > 60 ms		35 (5.0)		2 (0.4)		17 (4.1)		2 (0.5)

Data are expressed as number of patients with abnormality (%). DHA-PQP, dihydroartemisinin-piperaquine; PA, pyronaridine-artesunate; ASAQ, artesunate-amodiaquine; AL, artemether-lumefantrine; Δ, Change from baseline.

### Adverse events

Sixty two serious adverse events, including 9 deaths, occurred in the entire WANECAM study but none of them was related to proarrhythmia or possible signs or symptoms related to proarrhythmia such as fainting/syncope, palpitations, convulsions/seizures or chest pain. Extensive description of adverse events in the WANECAM study have been reported earlier^15^.

## Discussion

In the present study, the effects of 4 different ACTs on the ECG were compared as part of the WANECAM study which included several thousand patients with uncomplicated malaria. Irrespective of the QT interval correction method used, the study results indicate that, in those patients, treatment with DHA-PQP and ASAQ causes QTc prolongation whereas a mild to moderate prolongation was observed with AL and no prolongation was observed with PA.

It has been shown, both in healthy subjects and patients, that administration of DHA-PQP prolongs the QT interval in healthy adults and malaria patients although the extent of prolongation varied widely from 7 to 45.2 ms^11–14^. Recently, a clinical trial carried out in Cambodia with DHA-PQP was halted over a concern of QTc prolongation^16^. The observed QT interval prolongation is caused by PQP rather than DHA since administration of 4-aminoquinolines like piperaquine has long been known to be associated with QT interval prolongation^17^ and this was recently confirmed for piperaquine^18^.

The risk of proarrhythmia of ASAQ has been poorly studied. AQ being a 4-aminoquinoline, it was not unexpected to observe a QT prolonging effect in the present study. In a study of uncomplicated malaria where children were treated with ASAQ or AL and QT correction was not optimized, the mean change from baseline on day 3 was −11.5 ms for QTcB + 13.4 ms for QTcF with heart rate dropping from 119 to 80 bpm^19^. In that study, no correlation between plasma concentrations of desethyl-amodiaquine, the main active metabolite of AQ, and changes in QTc was found.

In addition to the present study, several reports have indicated a small QT prolonging effect following administration of AL of about 10 ms in both healthy subjects^12^ and malaria patients^19,20^. Cardiac safety was typically not included in several clinical trials with PA^21^. As part of two drug-drug interaction studies, ECGs were recorded as part of the safety assessments^22,23^. In these studies no effects of PA on the QT interval were apparent and this lack of an effect was confirmed in the present study.

Correction of the QT interval using the classical correction methods in this study did not result in satisfactory correction after treatment with Bazett under-correcting and Fridericia over-correcting QT and yielding quite different QTc changes from baseline. A study-specific or pre-dose data-driven correction, which has been proposed for studies involving children^24^ and which took into account both sex and age, performed better than Bazett’s and Fridericia’s correction methods but still was not optimal as evidenced by a significant slope of the ΔQTc versus ΔRR relationship. The large fall in heart rate observed in malaria patients receiving effective treatment^19,25^, contributes to the absence of adequate QT interval correction formula such as what we found in our study where none of the investigated correction methods properly corrected the QT interval after treatment. This decrease in heart rate was most likely the result of treatment of the disease rather than a direct effect of study treatments on heart rate since such an effect was not observed in healthy subjects^22,23,26,27^.

Since commonly used correction methods failed to obtain reliable results due to very different heart rates at baseline and on-treatment, a new approach was used to estimate drugs effect on QT interval. This approach consists of modeling the ΔQTc versus ΔRR relationship and estimating ΔQTc in the absence of a change in heart rate, i.e., change from baseline at ΔRR = 0. Using this correction-free and heart rate-free approach, marked QTc prolonging effects of DHA-PQP and ASAQ were noted while AL had a smaller effect and PA did not affect the QT interval. The QTc prolonging effects of DHA-PQP and AL, as assessed with the ΔQTc versus ΔRR relationship in patients with malaria, were comparable to the effects of these antimalarial drugs measured in a dedicated QT study performed in healthy volunteers^28^. The ΔQTc-RR0 method uses all available information from all subjects to estimate an average drug’s effect on the corrected QT interval at a given time point, which improves its accuracy as evidenced by its independence on the appropriateness of the correction formula being used (Table 4). The advantage of this method is that it can account for very different heart rates before and during drug exposure. Its disadvantage is that it cannot estimate the effect in individual subjects/patients.

In this particular study, only one post-baseline ECG was recorded and the maximum effect of study treatments on the QT interval may not have been captured. However, the ΔQTc-RR0 approach can also be applied in the more common situation of recording multiple ECGs at different time points after treatment.

Both study-specific correction of the QT interval and the ΔQTc-RR0 approach can only be used post-hoc, i.e., are not available to treating physicians at the bedside. It has been shown that in infants and young children Bazett’s correction provided the most consistent calculation of QTc^29^.

None of the adverse events reported in the study were related to proarrhythmia or possible signs or symptoms related to proarrhythmia, which is reassuring regarding the cardiac safety of the tested ACTs. However, because patients with baseline QTc > 450 ms were excluded and the sample size was relatively small to detect an event as rare as torsade de pointes, we cannot exclude a risk of proarrhythmia associated with ACTs which prolonged QTc. Recent data indicate that, despite its effect on ventricular repolarization, DHA-PQP is not associated with an increased risk of sudden death^30,31^. It is however advisable to continue cardiac monitoring for DHA-PQP and ASAQ because the amplitude of QTc prolongation with these drugs is large.

In conclusion, the present study indicates that treatment with DHA-PQP and ASAQ prolong QT interval whereas such an effect is less marked with AL and unlikely with PA. No clinical signs or symptoms of proarrhythmia with any treatment were observed during the study. Our data support the use of the intercept of the ΔQTc vs. ΔRR relationship to assess the true effects of antimalarials on the QT interval. This method also may apply to QT assessment with any drug when there are important variations of heart rate between off-drug and on-drug measurements.

## Methods

### Study population and design

The target population for this study included male and female patients with acute uncomplicated *Plasmodium* sp. malaria as evidenced by the presence of fever and by positive microscopy of *Plasmodium* sp. In all treatment groups, patients were aged 6 months or older, weighing ≥5 kg without severe malnutrition, were able to swallow study medication and agreed to remain under medical observation. However, in the PA group, the age and bodyweight of the first enrolled patients were ≥15 years and ≥24 kg. After safety review of the first 20 patients who were treated at least twice, the age and body weight limits were reduced to ≥2 years and ≥15 kg. These limits were reduced to 6 months or older and weighing ≥5 kg when 40 patients were treated at least twice with satisfactory safety. Patients could not participate in the study if they had severe or complicated malaria, severe vomiting or diarrhoea, known history or evidence of any clinically significant disorders, a QTc value (Bazett or Fridericia correction) of more than 450 ms, haemoglobin of less than 7 g/dL, non-malarial febrile conditions, known drug hypersensitivity, antimalarial treatment within the previous 2 weeks or an investigational drug within 4 weeks, known or suspected alcohol abuse, known HIV-antibody positivity, hepatitis A IgM, hepatitis B surface antigen or hepatitis C antibody, alanine aminotransferase concentration of more than twice the upper limit of normal (ULN), or significant renal impairment (creatinine >1.5 × ULN). The intake of an antimalarial agent or an investigational drug within 2 and 4 weeks prior to study start, respectively, was prohibited.

This was a phase IIIb/IV comparative, randomized, multi-center, open-label, parallel 3-arm clinical study. The study was conducted in 7 centers in 3 countries: 4 centers in Mali, 2 in Burkina Faso and 1 in the Republic of Guinea. In this study, the investigational ACTs PA and DHA-PQP were compared to AL or ASAQ depending on the clinical practice in each center. For each microscopically confirmed malaria episode, the artemisinin-based combination therapy was administered orally once-a-day (PA, DHA-PQP, ASAQ) or twice-a-day (AL) for 3 consecutive days. There was a minimum of 4 weeks required between different malaria episodes for a patient to be treated within this study. If less than 4 weeks had elapsed between episodes, rescue therapy was administered. Dosing of each treatment depended on the patient’s body weight and was done according to the respective labeling information.

The WANECAM study included 4,710 patients of whom 2,091 had ECG data before and during drug exposure during the first episode of malaria. Such a sample size allowed the detection of QTc increase of at least 4 ms at the 5% level (one-sided) with a power >90% for all treatment groups.

The Ethics Committee of each center and the competent national Health Authority approved the study protocol. Patient’s informed consent was obtained according to the ethical principles stated in the Declaration of Helsinki, the applicable guidelines for Good Clinical Practice, and the applicable local laws and regulations.

### ECG collection and handling

Digital single 12-lead ECGs were recorded using an ECG recorder (Cartouch, Cardionics, Brussels, Belgium) at the following time points: predose on the first day of treatment and 2 to 4 h after the last dose of treatment on Day 3. When deemed necessary by the investigator, other ECGs could be recorded but these were not considered for the current analyses. All ECGs were transferred to a central ECG laboratory (Cardiabase, Nancy, France) for reading. A computer-assisted, semi-automatic, on-screen measurement of the digital ECG waveforms was used for the reading (ECG Manager^©^, Cardiabase, Nancy, France). All interval measurements (PR, QRS, QT) were made from the superimposed median beats. Each median beat was mathematically derived from the available 10-second recording of the individual lead. The 12 individual median beats were graphically displayed as temporally aligned and overlapped ECG complexes. Global interval measurements were subsequently defined as the interval from the earliest onset observed on any of the 12 superimposed leads to the latest offset observed on any of the 12 leads. The RR interval was assessed automatically by the software from all the sinus rhythm complexes recorded over a 10-second period of the ECG.

Using the generic formula QTc = QT/(RR)^bi^, where b_i_ is a correction exponent, a number of different corrections were performed: QTc according to Bazett (QTcB), where b_i_ is 0.5; QTc according to Fridericia (QTcF), where b_i_ is 0.33, and a study-specific correction (QTcSS) where b_i_, estimated using pre-dose QT and RR values, was dependent on sex and age. For QTcSS, the following age groups were defined: <2 years, 2 to 11 years, 12 to 18 years and >18 years.

### Statistical analysis

Only those patients who had valid ECG recordings both at baseline and on Day 3 were included in the present analyses. Changes from baseline in ECG parameters on Day 3 of the first malaria episode period were compared between treatment arms using an analysis of variance model including fixed effects for treatment and country. An estimate of the treatment effect was calculated with its 90% confidence interval. Categorized age and country were also included in the model as covariate. The covariates sex and body weight were investigated graphically (data not shown).

For QTcSS determination the QT-RR relationship of pre-dose data was modelled using the following equation: Log (QT_ij_) = a_i_ + b_i_.log(RR_ij_) + e_ij_ where a_i_ is the intercept, b_i_ is the slope, corresponding to the power in the QTcSS formula, i is a grouping variable (gender, age group or both), j is the subject and eij is the residual error.

The adequacy of the QT correction methods for post-dose data was assessed using the following equation: ΔQTc_i_ = a + b•ΔRR_i_ + e_i_, where a is the intercept, b is the slope, i is the subject and e_i_ is the residual error. Adequacy of a QT correction method after treatment was assessed by plotting individual ΔQTc values versus corresponding ΔRR values and subsequent linear regression as determined using the equation. A correction accounting perfectly for variation in heart rate should display a horizontal a regression line. A heart rate correction-free estimate of the average QT change in the population (ΔQT-RR0) was obtained by estimating the intercept and its 90% confidence interval of the ΔQTc versus the ΔRR regression line, i.e., the QT change in the absence of RR change (RR0), using each correction formula.

The absolute number and frequency of abnormal QTc values was determined using the following categories: 450 ms < QTc ≤ 480 ms, 480 ms < QTc ≤ 500 ms and QTc interval >500 ms for absolute values and 30 ms < QTc ≤ 60 ms and QTc interval >60 ms for changes from baseline.
